# (1R*,2R*,4S*,5R*,6R*,8S*)-4,8-Dimethyl-2,6-diphenyl­bicyclo­[3.3.1]nonane-2,6-diol

**DOI:** 10.1107/S1600536809009933

**Published:** 2009-03-28

**Authors:** Vi T. Nguyen, Roger Bishop, Donald C. Craig, Marcia L. Scudder

**Affiliations:** a School of Chemistry, University of New South Wales, Sydney 2052, Australia

## Abstract

The racemic title compound, C_23_H_28_O_2_, crystallizes in the space group *C*2/*c* as a layered structure in which a centrosymmetric three hydrogen bond sequence links four molecules. Both hydroxy groups are involved in this arrangement, but they differ in that one participates in two hydrogen bonds while the other takes part in only one. Between layers, the aromatic rings take part in edge-face interactions [shortest C—H⋯C distances 3.04, 3.10 and 3.12 Å and angle between normal to planes 86.7(2)°], forming a centrosymmetric dimer. The lattice is further stabilized by C—H⋯π interactions involving both methyl (shortest C⋯C 3.82 and 3.97 Å) and methylene (shortest C⋯C 3.60 Å) groups.

## Related literature

Phenyl­ation of *endo*-4, *endo*-8-dimethyl­bicyclo­[3.3.1]nonane-2,6- dione (Kim *et al.*, 2002 ▶) occurs selectively on the *exo*-faces of the V-shaped mol­ecule to yield the title compound. The related 2,6-dimethyl- substituted compound (Nguyen *et al.*, 2001*b* ▶) crystallizes with a hydrogen-bonded ladder structure (Nguyen *et al.*, 2001*a* ▶) that is very different to the pattern reported here.
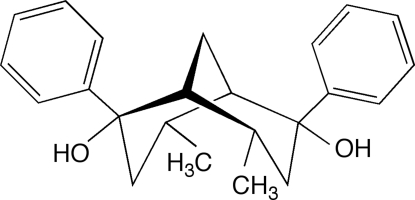


## Experimental

### 

#### Crystal data

C_23_H_28_O_2_
*M* *_r_* = 336.5
Monoclinic, 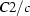

*a* = 18.462 (4) Å
*b* = 13.310 (1) Å
*c* = 14.824 (3) Å
β = 92.92 (1)°
*V* = 3638 (1) Å^3^
*Z* = 8
Cu *K*α radiation
μ = 0.59 mm^−1^
*T* = 294 K
0.30 × 0.15 × 0.12 mm

#### Data collection

Enraf–Nonius CAD-4 diffractometer
Absorption correction: none
3585 measured reflections
3442 independent reflections
2292 reflections with *I* > 2σ(*I*)
*R*_int_ = 0.034
1 standard reflections frequency: 30 min intensity decay: 4%

#### Refinement

*R*[*F* ^2^ > 2σ(*F* ^2^)] = 0.045
*wR*(*F* ^2^) = 0.062
*S* = 1.59
3442 reflections
160 parameters
H-atom parameters not refined
Δρ_max_ = 0.31 e Å^−3^
Δρ_min_ = −0.34 e Å^−3^

### 

Data collection: *CAD-4 Manual* (Schagen *et al.*, 1989 ▶); cell refinement: *CAD-4 Manual*; data reduction: local program; program(s) used to solve structure: *SIR92* (Altomare *et al.*, 1994 ▶); program(s) used to refine structure: *RAELS* (Rae, 2000 ▶); molecular graphics: *ORTEP-3* (Farrugia, 1997 ▶) and *CrystalMaker* (CrystalMaker, 2005 ▶); software used to prepare material for publication: local programs.

## Supplementary Material

Crystal structure: contains datablocks global, I. DOI: 10.1107/S1600536809009933/kp2208sup1.cif

Structure factors: contains datablocks I. DOI: 10.1107/S1600536809009933/kp2208Isup2.hkl

Additional supplementary materials:  crystallographic information; 3D view; checkCIF report

## Figures and Tables

**Table 1:** Hydrogen-bond geometry (Å, °)

*D*—H⋯*A*	*D*—H	H⋯*A*	*D*⋯*A*	*D*—H⋯*A*
O1—H1*O*1⋯O1^i^	1.00	1.97	2.943 (2)	163
O1—H1′*O*1⋯O2^ii^	1.00	2.04	2.935 (2)	148
O2—H1*O*2⋯O1^iii^	1.00	1.95	2.935 (2)	169

Symmetry codes: (i) 
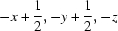
; (ii) 
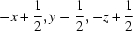
; (iii) 
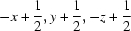
.

**Fig. 1.**
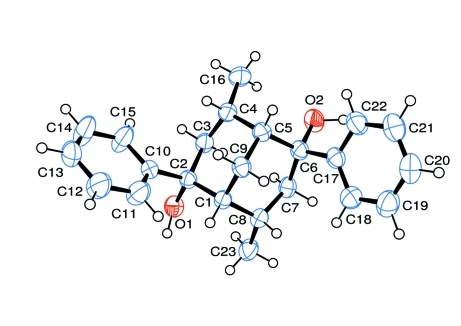
Molecular structure of the compound, with ellipsoids drawn at 50% probability level. Only one of the two disordered hydrogen positions is shown for each hydroxy group.

**Fig. 2.**
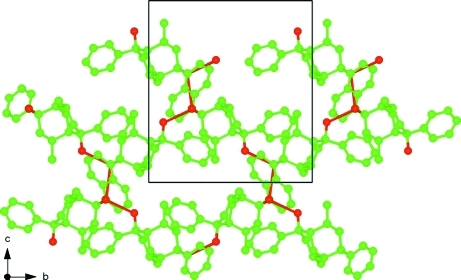
A hydrogen bonded layer in the bc plane. Hydrogen bonds are shown by red lines. In Figs. 2 and 3 C is green, O is red.

**Fig. 3.**
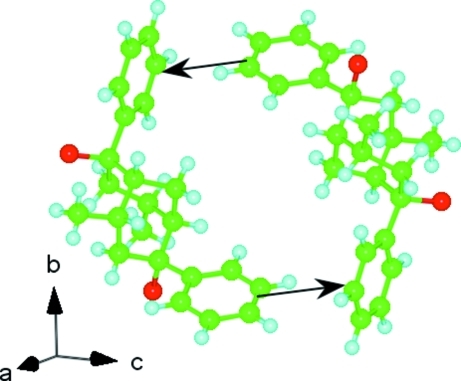
The centrosymmetric dimer links layers by a double edge-face interactions (shown as black arrows).

## supplementary crystallographic information

### Comment

Molecules (Fig. 1) are linked by a centrosymmetric triplet of hydrogen bonds utilizing one hydroxy group from each of four molecules (Table 1). The other hydroxy group of each of these molecules participates in an identical hydogen bonding unit, leading to a layer structure in the *bc* plane (Fig. 2). The hydrogen bonding in this compound is somewhat unusual in that hydroxy group, O1, participates in two hydrogen bonds, whereas O2 only participates in one. The majority of the alicyclic diols studied by us (Kim *et al.*, 2002) have one donor and one acceptor hydrogen bond for each hydroxy group.

The pendant phenyl rings do not participate in the common aromatic offset face-to-face interaction in the crystal structure. Instead, one phenyl ring (C10→C15) is the acceptor of two C_methyl_—H···π interactions, one to each surface of the ring (C16—H···π, C23—H···π with shortest H···C distances of 3.97 and 3.82 Å, respectively). The second phenyl ring (C17→C22) takes part in a C_methylene_—H···π interaction on one surface (C3—H···π with a shortest H···C distance of 3.60 Å). Its second surface is the acceptor of an edge-to-face interaction utilizing the edge of the C10→C15 ring and creating a centrosymmetric dimer between layers (Fig. 3).

### Experimental

A solution of racemic *endo*-4,*endo*-8-dimethylbicyclo[3.3.1]nonane-2,6-dione (Kim *et al.*, 2002) (0.79 g, 4.4 mmol) in dry tetrahydrofuran (30 ml) was added dropwise to a stirred solution of excess phenylmagnesium bromide in dry diethyl ether (10 ml) at -10°C. After 12 h at rt, the reaction was subjected to a standard Grignard reaction work-up to yield the title compound (0.67 g, 47%), m.p. 438-440 K (from acetonitrile). Found: C 82.15, H 8.34; C_23_H_28_O_2_ requires C 82.10, H 8.39%. ^13^C NMR (75.5 MHz, CDCl_3_) δ: 22.0 (CH_3_), 32.5 (CH_2_), 36.2 (CH), 42.4 (CH), 42.6 (CH_2_), 78.4 (C), 125.5 (CH), 127.0 (CH), 128.2 (CH), 148.3 (C). ^1^H NMR (300 MHz, CDCl_3_) δ: 1.18 (t, *J*=3.0 Hz, 2H), 1.46 (d, *J*=6.4 Hz, 6H), 1.57 (bs, 2H, exchanged with D_2_O), 2.18–2.39 (m, 6H), 2.44–2.47 (m, 2H), 7.22–7.27 (m, 2H), 7.31–7.36 (m, 4H), 7.52–7.55 (m, 4H). X-ray quality crystals were obtained from tetrahydrofuran solution.

### Refinement

The hydrogen atoms on the hydroxy groups are disordered over two sites of equal occupancy. This is a requirement of the centrosymmetric hydrogen bonding arrangement found in the lattice. The hydroxy hydrogen atoms were located on a difference map, and were then fixed at a position along the OH vector with O—H = 1.0 Å. Hydrogen atoms attached to C were included at calculated positions (C—H = 1.0 Å). All hydrogen atoms were refined with isotropic thermal parameters equivalent to those of the atom to which they were bonded.

### Crystal data

**Table:**

C_23_H_28_O_2_	*F*(000) = 1456.0
*M**_r_* = 336.5	*D*_x_ = 1.23 Mg m^−^^3^
Monoclinic, *C*2/*c*	Cu *K*α radiation, λ = 1.54184 Å
*a* = 18.462 (4) Å	Cell parameters from 10 reflections
*b* = 13.310 (1) Å	θ = 20–23°
*c* = 14.824 (3) Å	µ = 0.59 mm^−^^1^
β = 92.92 (1)°	*T* = 294 K
*V* = 3638 (1) Å^3^	Prism, colourless
*Z* = 8	0.30 × 0.15 × 0.12 mm

### Data collection

**Table:**

Enraf–Nonius CAD-4 diffractometer	θ_max_ = 70°
ω–2θ scans	*h* = −22→22
3585 measured reflections	*k* = 0→16
3442 independent reflections	*l* = −18→0
2292 reflections with *I* > 2σ(*I*)	1 standard reflections every 30 min
*R*_int_ = 0.034	intensity decay: 4%

### Refinement

**Table:**

Refinement on *F*	0 restraints
*R*[*F*^2^ > 2σ(*F*^2^)] = 0.045	H-atom parameters not refined
*wR*(*F*^2^) = 0.062	*w* = 1/[σ^2^(*F*) + 0.0004*F*^2^]
*S* = 1.59	(Δ/σ)_max_ = 0.004
3442 reflections	Δρ_max_ = 0.31 e Å^−^^3^
160 parameters	Δρ_min_ = −0.34 e Å^−^^3^

### Fractional atomic coordinates and isotropic or equivalent isotropic displacement parameters (Å^2^)

**Table:**

	*x*	*y*	*z*	*U*_iso_*/*U*_eq_	Occ. (<1)
O1	0.27017 (6)	0.26370 (9)	0.09652 (8)	0.0465 (3)	
O2	0.30304 (7)	0.58902 (10)	0.32886 (8)	0.0550 (4)	
C1	0.32906 (9)	0.42870 (12)	0.08333 (11)	0.0350 (4)	
C2	0.33263 (9)	0.32328 (12)	0.12682 (11)	0.0372 (4)	
C3	0.3302 (1)	0.3328 (1)	0.2293 (1)	0.0435 (4)	
C4	0.3858 (1)	0.4068 (1)	0.2711 (1)	0.0469 (5)	
C5	0.38388 (9)	0.51069 (14)	0.22386 (11)	0.0409 (4)	
C6	0.31736 (9)	0.58053 (13)	0.23431 (11)	0.0406 (4)	
C7	0.24945 (9)	0.53770 (13)	0.18446 (12)	0.0426 (4)	
C8	0.25972 (9)	0.49329 (13)	0.09061 (12)	0.0405 (4)	
C9	0.39285 (9)	0.49236 (13)	0.12283 (12)	0.0400 (4)	
C10	0.39953 (8)	0.26407 (10)	0.09682 (8)	0.0401 (4)	
C11	0.42900 (8)	0.28028 (12)	0.01427 (10)	0.0643 (4)	
C12	0.48531 (8)	0.22117 (14)	−0.01466 (10)	0.0737 (6)	
C13	0.51343 (9)	0.14468 (12)	0.03804 (10)	0.0615 (6)	
C14	0.48475 (9)	0.12773 (11)	0.12005 (12)	0.0781 (6)	
C15	0.42844 (8)	0.18666 (12)	0.14924 (10)	0.0664 (5)	
C16	0.3837 (1)	0.4072 (2)	0.3743 (1)	0.0667 (6)	
C17	0.33675 (7)	0.68703 (11)	0.20411 (8)	0.0424 (4)	
C18	0.30590 (7)	0.73420 (11)	0.12855 (9)	0.0548 (5)	
C19	0.32341 (9)	0.83264 (11)	0.10750 (10)	0.0642 (6)	
C20	0.37202 (8)	0.88615 (12)	0.16127 (10)	0.0625 (6)	
C21	0.40304 (8)	0.84036 (11)	0.23640 (11)	0.0647 (5)	
C22	0.38568 (8)	0.74193 (11)	0.25771 (9)	0.0542 (5)	
C23	0.1887 (1)	0.4463 (2)	0.0533 (1)	0.0565 (5)	
H1O1	0.2639	0.2649	0.0291	0.047	0.5
H1'O1	0.2614	0.2068	0.1386	0.047	0.5
H1O2	0.2833	0.6535	0.3521	0.055	0.5
H1'O2	0.2723	0.5320	0.3486	0.055	0.5
HC1	0.3367	0.4197	0.0175	0.035	
H1C3	0.3397	0.2651	0.2566	0.043	
H2C3	0.2807	0.3560	0.2440	0.043	
HC4	0.4341	0.3777	0.2583	0.047	
HC5	0.4277	0.5486	0.2473	0.041	
H1C7	0.2131	0.5932	0.1775	0.043	
H2C7	0.2299	0.4834	0.2230	0.043	
HC8	0.2674	0.5528	0.0510	0.041	
H1C9	0.3937	0.5583	0.0906	0.040	
H2C9	0.4394	0.4559	0.1146	0.040	
HC11	0.4094	0.3357	−0.0255	0.106	
HC12	0.5056	0.2347	−0.0748	0.121	
HC13	0.5540	0.1022	0.0171	0.074	
HC14	0.5046	0.0722	0.1594	0.130	
HC15	0.4084	0.1728	0.2094	0.108	
H1C16	0.4204	0.4560	0.4001	0.067	
H2C16	0.3343	0.4276	0.3921	0.067	
H3C16	0.3951	0.3384	0.3981	0.067	
HC18	0.2702	0.6967	0.0882	0.071	
HC19	0.3002	0.8649	0.0524	0.086	
HC20	0.3846	0.9570	0.1460	0.074	
HC21	0.4387	0.8783	0.2765	0.088	
HC22	0.4091	0.7102	0.3130	0.069	
H1C23	0.1959	0.4176	−0.0079	0.056	
H2C23	0.1737	0.3917	0.0948	0.056	
H3C23	0.1501	0.4990	0.0486	0.056	

### Atomic displacement parameters (Å^2^)

**Table:**

	*U*^11^	*U*^22^	*U*^33^	*U*^12^	*U*^13^	*U*^23^
O1	0.0467 (7)	0.0394 (7)	0.0538 (8)	−0.0053 (6)	0.0064 (6)	0.0009 (6)
O2	0.079 (1)	0.0515 (8)	0.0364 (7)	0.0074 (7)	0.0167 (6)	−0.0026 (6)
C1	0.0390 (9)	0.0344 (9)	0.0321 (9)	0.0031 (7)	0.0066 (7)	0.0017 (7)
C2	0.0421 (9)	0.0350 (9)	0.0347 (9)	0.0021 (7)	0.0049 (7)	0.0004 (7)
C3	0.056 (1)	0.040 (1)	0.0345 (9)	0.0080 (8)	0.0085 (8)	0.0048 (8)
C4	0.057 (1)	0.047 (1)	0.036 (1)	0.0145 (9)	−0.0012 (8)	−0.0018 (8)
C5	0.0410 (9)	0.043 (1)	0.039 (1)	0.0035 (8)	0.0019 (7)	−0.0040 (8)
C6	0.047 (1)	0.041 (1)	0.0351 (9)	0.0058 (8)	0.0078 (7)	−0.0021 (8)
C7	0.041 (1)	0.0391 (9)	0.049 (1)	0.0048 (8)	0.0104 (8)	−0.0010 (8)
C8	0.042 (1)	0.0359 (9)	0.044 (1)	0.0035 (8)	0.0024 (7)	0.0029 (8)
C9	0.0383 (9)	0.041 (1)	0.041 (1)	0.0027 (8)	0.0072 (7)	−0.0030 (8)
C10	0.0461 (6)	0.0363 (7)	0.0374 (6)	0.0049 (5)	−0.0017 (5)	−0.0050 (5)
C11	0.0687 (7)	0.0782 (9)	0.0472 (7)	0.0294 (7)	0.0144 (6)	0.0092 (5)
C12	0.0726 (8)	0.096 (1)	0.0541 (8)	0.0348 (8)	0.0148 (7)	−0.0006 (7)
C13	0.0570 (7)	0.0582 (9)	0.069 (1)	0.0159 (7)	0.0054 (6)	−0.0150 (7)
C14	0.0783 (9)	0.0679 (9)	0.090 (1)	0.0373 (8)	0.0233 (8)	0.0189 (7)
C15	0.0716 (7)	0.0639 (8)	0.0652 (7)	0.0319 (6)	0.0168 (6)	0.0192 (6)
C16	0.097 (2)	0.066 (2)	0.036 (1)	0.022 (1)	−0.010 (1)	0.002 (1)
C17	0.0439 (7)	0.0426 (5)	0.0411 (7)	0.0037 (5)	0.0076 (5)	−0.0070 (4)
C18	0.0647 (8)	0.0496 (5)	0.0497 (7)	0.0024 (5)	0.0012 (5)	0.0020 (5)
C19	0.078 (1)	0.0509 (6)	0.0642 (8)	0.0039 (5)	0.0095 (6)	0.0075 (6)
C20	0.070 (1)	0.0448 (5)	0.074 (1)	0.0009 (5)	0.0224 (8)	−0.0018 (5)
C21	0.070 (1)	0.0486 (5)	0.075 (1)	−0.0098 (6)	0.0067 (7)	−0.0079 (5)
C22	0.0575 (8)	0.0477 (5)	0.0571 (7)	−0.0055 (5)	0.0001 (5)	−0.0075 (5)
C23	0.046 (1)	0.048 (1)	0.074 (2)	0.0075 (9)	−0.010 (1)	−0.004 (1)

### Geometric parameters (Å, °)

**Table:**

O1—C2	1.452 (2)	C9—H2C9	1.000
O1—H1O1	1.000	C10—C11	1.381 (1)
O1—H1'O1	1.000	C10—C15	1.381 (1)
O2—C6	1.444 (2)	C11—C12	1.389 (1)
O2—H1O2	1.000	C11—HC11	1.000
O2—H1'O2	1.000	C12—C13	1.369 (1)
C1—C2	1.544 (2)	C12—HC12	1.000
C1—C8	1.550 (2)	C13—C14	1.369 (1)
C1—C9	1.542 (2)	C13—HC13	1.000
C1—HC1	1.000	C14—C15	1.389 (1)
C2—C3	1.527 (2)	C14—HC14	1.000
C2—C10	1.549 (2)	C15—HC15	1.000
C3—C4	1.531 (3)	C16—H1C16	1.000
C3—H1C3	1.000	C16—H2C16	1.000
C3—H2C3	1.000	C16—H3C16	1.000
C4—C5	1.550 (2)	C17—C18	1.381 (1)
C4—C16	1.532 (3)	C17—C22	1.381 (1)
C4—HC4	1.000	C18—C19	1.389 (1)
C5—C6	1.554 (2)	C18—HC18	1.000
C5—C9	1.535 (2)	C19—C20	1.369 (1)
C5—HC5	1.000	C19—HC19	1.000
C6—C7	1.532 (2)	C20—C21	1.369 (1)
C6—C17	1.534 (2)	C20—HC20	1.000
C7—C8	1.532 (2)	C21—C22	1.389 (1)
C7—H1C7	1.000	C21—HC21	1.000
C7—H2C7	1.000	C22—HC22	1.000
C8—C23	1.530 (2)	C23—H1C23	1.000
C8—HC8	1.000	C23—H2C23	1.000
C9—H1C9	1.000	C23—H3C23	1.000
			
C2—O1—H1O1	110.4	C1—C9—C5	109.8 (1)
C2—O1—H1'O1	111.8	C1—C9—H1C9	109.4
H1O1—O1—H1'O1	128.5	C1—C9—H2C9	109.4
C6—O2—H1O2	119.3	C5—C9—H1C9	109.4
C6—O2—H1'O2	111.1	C5—C9—H2C9	109.4
H1O2—O2—H1'O2	109.2	H1C9—C9—H2C9	109.5
C2—C1—C8	119.5 (1)	C2—C10—C11	122.0 (1)
C2—C1—C9	109.0 (1)	C2—C10—C15	120.6 (1)
C2—C1—HC1	107.0	C11—C10—C15	117.2 (1)
C8—C1—C9	106.6 (1)	C10—C11—C12	121.3 (1)
C8—C1—HC1	107.0	C10—C11—HC11	119.3
C9—C1—HC1	107.0	C12—C11—HC11	119.3
O1—C2—C1	110.6 (1)	C11—C12—C13	120.9 (1)
O1—C2—C3	106.9 (1)	C11—C12—HC12	119.6
O1—C2—C10	105.4 (1)	C13—C12—HC12	119.6
C1—C2—C3	109.7 (1)	C12—C13—C14	118.5 (1)
C1—C2—C10	111.1 (1)	C12—C13—HC13	120.8
C3—C2—C10	113.1 (1)	C14—C13—HC13	120.8
C2—C3—C4	113.8 (2)	C13—C14—C15	120.9 (1)
C2—C3—H1C3	108.4	C13—C14—HC14	119.6
C2—C3—H2C3	108.4	C15—C14—HC14	119.6
C4—C3—H1C3	108.4	C10—C15—C14	121.3 (1)
C4—C3—H2C3	108.4	C10—C15—HC15	119.3
H1C3—C3—H2C3	109.5	C14—C15—HC15	119.3
C3—C4—C5	113.0 (1)	C4—C16—H1C16	109.5
C3—C4—C16	110.9 (2)	C4—C16—H2C16	109.5
C3—C4—HC4	105.1	C4—C16—H3C16	109.5
C5—C4—C16	116.5 (2)	H1C16—C16—H2C16	109.5
C5—C4—HC4	105.1	H1C16—C16—H3C16	109.5
C16—C4—HC4	105.1	H2C16—C16—H3C16	109.5
C4—C5—C6	119.3 (2)	C6—C17—C18	124.3 (1)
C4—C5—C9	107.4 (1)	C6—C17—C22	118.4 (1)
C4—C5—HC5	107.1	C18—C17—C22	117.2 (1)
C6—C5—C9	108.5 (1)	C17—C18—C19	121.3 (1)
C6—C5—HC5	107.1	C17—C18—HC18	119.3
C9—C5—HC5	107.1	C19—C18—HC18	119.3
O2—C6—C5	109.2 (1)	C18—C19—C20	120.9 (1)
O2—C6—C7	108.1 (1)	C18—C19—HC19	119.6
O2—C6—C17	105.5 (1)	C20—C19—HC19	119.6
C5—C6—C7	111.1 (1)	C19—C20—C21	118.5 (1)
C5—C6—C17	109.0 (1)	C19—C20—HC20	120.8
C7—C6—C17	113.6 (1)	C21—C20—HC20	120.8
C6—C7—C8	116.4 (1)	C20—C21—C22	120.9 (1)
C6—C7—H1C7	107.7	C20—C21—HC21	119.6
C6—C7—H2C7	107.7	C22—C21—HC21	119.6
C8—C7—H1C7	107.7	C17—C22—C21	121.3 (1)
C8—C7—H2C7	107.7	C17—C22—HC22	119.3
H1C7—C7—H2C7	109.5	C21—C22—HC22	119.3
C1—C8—C7	114.7 (1)	C8—C23—H1C23	109.5
C1—C8—C23	116.3 (1)	C8—C23—H2C23	109.5
C1—C8—HC8	104.8	C8—C23—H3C23	109.5
C7—C8—C23	110.0 (2)	H1C23—C23—H2C23	109.5
C7—C8—HC8	104.8	H1C23—C23—H3C23	109.5
C23—C8—HC8	104.8	H2C23—C23—H3C23	109.5
			
H1O1—O1—C2—C1	50.8	C4—C5—C9—C1	62.1 (2)
H1O1—O1—C2—C3	170.1	C4—C5—C9—H1C9	−177.9
H1O1—O1—C2—C10	−69.4	C4—C5—C9—H2C9	−58.0
H1'O1—O1—C2—C1	−159.4	C6—C5—C9—C1	−68.1 (2)
H1'O1—O1—C2—C3	−40.1	C6—C5—C9—H1C9	52.0
H1'O1—O1—C2—C10	80.5	C6—C5—C9—H2C9	171.9
H1O2—O2—C6—C5	146.9	HC5—C5—C9—C1	176.7
H1O2—O2—C6—C7	−92.1	HC5—C5—C9—H1C9	−63.2
H1O2—O2—C6—C17	29.8	HC5—C5—C9—H2C9	56.7
H1'O2—O2—C6—C5	−84.8	O2—C6—C7—C8	−162.2 (1)
H1'O2—O2—C6—C7	36.2	O2—C6—C7—H1C7	76.8
H1'O2—O2—C6—C17	158.1	O2—C6—C7—H2C7	−41.2
C8—C1—C2—O1	52.7 (2)	C5—C6—C7—C8	−42.4 (2)
C8—C1—C2—C3	−64.9 (2)	C5—C6—C7—H1C7	−163.4
C8—C1—C2—C10	169.3 (1)	C5—C6—C7—H2C7	78.6
C9—C1—C2—O1	175.5 (1)	C17—C6—C7—C8	81.0 (2)
C9—C1—C2—C3	57.9 (2)	C17—C6—C7—H1C7	−40.0
C9—C1—C2—C10	−67.8 (2)	C17—C6—C7—H2C7	−158.0
HC1—C1—C2—O1	−69.0	O2—C6—C17—C18	−130.1 (1)
HC1—C1—C2—C3	173.4	O2—C6—C17—C22	46.7 (1)
HC1—C1—C2—C10	47.6	C5—C6—C17—C18	112.8 (1)
C2—C1—C8—C7	73.1 (2)	C5—C6—C17—C22	−70.4 (1)
C2—C1—C8—C23	−57.3 (2)	C7—C6—C17—C18	−11.8 (2)
C2—C1—C8—HC8	−172.5	C7—C6—C17—C22	165.0 (1)
C9—C1—C8—C7	−50.9 (2)	C6—C7—C8—C1	42.0 (2)
C9—C1—C8—C23	178.7 (1)	C6—C7—C8—C23	175.4 (1)
C9—C1—C8—HC8	63.5	C6—C7—C8—HC8	−72.4
HC1—C1—C8—C7	−165.2	H1C7—C7—C8—C1	163.0
HC1—C1—C8—C23	64.4	H1C7—C7—C8—C23	−63.6
HC1—C1—C8—HC8	−50.8	H1C7—C7—C8—HC8	48.6
C2—C1—C9—C5	−65.5 (2)	H2C7—C7—C8—C1	−79.0
C2—C1—C9—H1C9	174.5	H2C7—C7—C8—C23	54.4
C2—C1—C9—H2C9	54.6	H2C7—C7—C8—HC8	166.6
C8—C1—C9—C5	64.8 (2)	C1—C8—C23—H1C23	−47.4
C8—C1—C9—H1C9	−55.3	C1—C8—C23—H2C23	72.6
C8—C1—C9—H2C9	−175.1	C1—C8—C23—H3C23	−167.4
HC1—C1—C9—C5	179.1	C7—C8—C23—H1C23	−180.0
HC1—C1—C9—H1C9	59.0	C7—C8—C23—H2C23	−60.0
HC1—C1—C9—H2C9	−60.9	C7—C8—C23—H3C23	60.0
O1—C2—C3—C4	−171.2 (1)	HC8—C8—C23—H1C23	67.8
O1—C2—C3—H1C3	68.1	HC8—C8—C23—H2C23	−172.2
O1—C2—C3—H2C3	−50.6	HC8—C8—C23—H3C23	−52.2
C1—C2—C3—C4	−51.4 (2)	C2—C10—C11—C12	−174.8 (1)
C1—C2—C3—H1C3	−172.0	C2—C10—C11—HC11	5.2
C1—C2—C3—H2C3	69.3	C15—C10—C11—C12	0.0 (0)
C10—C2—C3—C4	73.3 (2)	C15—C10—C11—HC11	180.0
C10—C2—C3—H1C3	−47.4	C2—C10—C15—C14	174.9 (1)
C10—C2—C3—H2C3	−166.1	C2—C10—C15—HC15	−5.1
O1—C2—C10—C11	90.9 (1)	C11—C10—C15—C14	0.0 (0)
O1—C2—C10—C15	−83.7 (1)	C11—C10—C15—HC15	180.0
C1—C2—C10—C11	−28.9 (2)	C10i—C11i—C12i—C13i	0.0 (0)
C1—C2—C10—C15	156.5 (1)	C10i—C11i—C12i—HC12i	−180.0
C3—C2—C10—C11	−152.7 (1)	HC11i—C11i—C12i—C13i	180.0
C3—C2—C10—C15	32.6 (2)	HC11i—C11i—C12i—HC12i	0.0
C2—C3—C4—C5	50.8 (2)	C11i—C12i—C13i—C14i	0.0 (0)
C2—C3—C4—C16	−176.2 (2)	C11i—C12i—C13i—HC13i	180.0
C2—C3—C4—HC4	−63.2	HC12i—C12i—C13i—C14i	180.0
H1C3—C3—C4—C5	171.4	HC12i—C12i—C13i—HC13i	0.0
H1C3—C3—C4—C16	−55.6	C12i—C13i—C14i—C15i	0.0 (0)
H1C3—C3—C4—HC4	57.4	C12i—C13i—C14i—HC14i	180.0
H2C3—C3—C4—C5	−69.9	HC13i—C13i—C14i—C15i	−180.0
H2C3—C3—C4—C16	63.1	HC13i—C13i—C14i—HC14i	0.0
H2C3—C3—C4—HC4	176.1	C13i—C14i—C15i—C10i	0.0 (0)
C3—C4—C5—C6	69.4 (2)	C13i—C14i—C15i—HC15i	−180.0
C3—C4—C5—C9	−54.4 (2)	HC14i—C14i—C15i—C10i	180.0
C3—C4—C5—HC5	−169.1	HC14i—C14i—C15i—HC15i	0.0
C16—C4—C5—C6	−60.9 (2)	C6i—C17i—C18i—C19i	176.8 (1)
C16—C4—C5—C9	175.3 (2)	C6i—C17i—C18i—HC18i	−3.2
C16—C4—C5—HC5	60.7	C22i—C17i—C18i—C19i	0.0 (0)
HC4—C4—C5—C6	−176.6	C22i—C17i—C18i—HC18i	180.0
HC4—C4—C5—C9	59.6	C6i—C17i—C22i—C21i	−177.0 (1)
HC4—C4—C5—HC5	−55.1	C6i—C17i—C22i—HC22i	3.0
C3—C4—C16—H1C16	180.0	C18i—C17i—C22i—C21i	0.0 (0)
C3—C4—C16—H2C16	−60.0	C18i—C17i—C22i—HC22i	180.0
C3—C4—C16—H3C16	60.0	C17i—C18i—C19i—C20i	0.0 (0)
C5—C4—C16—H1C16	−48.8	C17i—C18i—C19i—HC19i	180.0
C5—C4—C16—H2C16	71.2	HC18i—C18i—C19i—C20i	−180.0
C5—C4—C16—H3C16	−168.8	HC18i—C18i—C19i—HC19i	0.0
HC4—C4—C16—H1C16	67.0	C18i—C19i—C20i—C21i	0.0 (0)
HC4—C4—C16—H2C16	−173.0	C18i—C19i—C20i—HC20i	−180.0
HC4—C4—C16—H3C16	−53.0	HC19i—C19i—C20i—C21i	180.0
C4—C5—C6—O2	50.1 (2)	HC19i—C19i—C20i—HC20i	0.0
C4—C5—C6—C7	−69.1 (2)	C19i—C20i—C21i—C22i	0.0 (0)
C4—C5—C6—C17	165.0 (1)	C19i—C20i—C21i—HC21i	−180.0
C9—C5—C6—O2	173.4 (1)	HC20i—C20i—C21i—C22i	−180.0
C9—C5—C6—C7	54.2 (2)	HC20i—C20i—C21i—HC21i	0.0
C9—C5—C6—C17	−71.8 (2)	C20i—C21i—C22i—C17i	0.0 (0)
HC5—C5—C6—O2	−71.4	C20i—C21i—C22i—HC22i	180.0
HC5—C5—C6—C7	169.4	HC21i—C21i—C22i—C17i	180.0
HC5—C5—C6—C17	43.4	HC21i—C21i—C22i—HC22i	0.0

### Hydrogen-bond geometry (Å, °)

**Table:**

*D*—H···*A*	*D*—H	H···*A*	*D*···*A*	*D*—H···*A*
O1—H1O1···O1^i^	1.00	1.97	2.943 (2)	163
O1—H1'O1···O2^ii^	1.00	2.04	2.935 (2)	148
O2—H1O2···O1^iii^	1.00	1.95	2.935 (2)	169

Symmetry codes: (i) −*x*+1/2, −*y*+1/2, −*z*; (ii) −*x*+1/2, *y*−1/2, −*z*+1/2; (iii) −*x*+1/2, *y*+1/2, −*z*+1/2.
